# In the Wake of COVID-19: The Developmental and Mental Health Fallout Amongst South African University Students

**DOI:** 10.1177/21676968231180960

**Published:** 2023-06-21

**Authors:** Katherine Bain, Tasneem Hassem, Nabeelah Bemath, Victor de Andrade, Sumaya Laher

**Affiliations:** 1 School of Human and Community Development, 37707 University of the Witwatersrand; 2 School of Anatomical Sciences, University of the Witwatersrand

**Keywords:** COVID-19, emerging adulthood, depression, social anxiety, delayed social skills acquisition

## Abstract

Multiple studies have noted the impacts on student mental health of the COVID-19 pandemic, associated national lockdowns and emergency remote teaching. In light of COVID-19 shifting from pandemic to endemic status, this study investigates the developmental and mental health consequences of the pandemic for a group of South African undergraduate students. A qualitative design allowed for the thematic analysis of the narratives of 140 humanities students, gathered through an online survey. This paper presents the ‘voices’ of this group to convey the intensity of their COVID-19 experience. The results suggest a loss of a sense of freedom and opportunities to explore and experiment, high levels of depression with a notable sense of hopelessness regarding the future and decreased motivation, and significant reports of social anxiety related to delays in the development of social skills due to social isolation, particular to the first-year cohort.

## Introduction

Students registered for courses in tertiary educational institutions make up approximately 33% of those aged 18–24 in South Africa. Much emphasis has been placed on providing greater access to higher education in order to foster skills development, in order to address historic racially-based, socioeconomic imbalances caused by Apartheid (Ajoodha et al., 2020). These students often represent the hopes of their families for an improved quality of life, with roughly 70% of these students being the first generation to be afforded an opportunity to attend university (Letseka & Maile, 2008). However, South African students have also been identified as an at-risk population, with (Bantjes et al., 2019, 2021).

Given the pre-existing mental health risks associated with the increased stress of tertiary level study, the mental health of university students during the height of the COVID-19 pandemic was of concern, in South Africa and internationally (Bantjes et al., 2019; Buizza et al., 2022; Lipson et al., 2019). Studies globally found increased levels of psychological distress within student samples (Chen & Lucock, 2022; Copeland et al., 2021; Laher et al., 2021; Li et al., 2021). A systematic review of studies on students from G20 countries found that life disruption, social distancing, and self-isolation due to the lockdown and quarantine requirements, were experienced as stressful and detrimental to students’ health and well-being (Nurrunnabi et al., 2020). Studies on South African students concurred with these findings (Laher et al., 2021; Visser & Law-van Wyk, 2021).

However, less is known regarding the consequences of this reported distress amongst youth post the COVID-19 pandemic (Farris et al., 2021). This study aimed to explore the persistence of COVID-19 related distress and perceived consequences of the pandemic amongst a cohort of South African students, contributing to understandings of the potential developmental costs of the pandemic for this already at-risk group of emerging adults. Differences and similarities between the year groups’ narratives were also explored in order to determine if the stage of the pandemic may have influenced student experience.

African populations are generally under-represented within international literature, so this study also hopes to contribute both to the documentation of South African university students’ experiences to the COVID-19 pandemic. The utility of the theory of emerging adulthood in aiding our understanding of this group of South African students’ perceptions of the consequences of the pandemic on their experiences and development evidences the theory’s potential applicability to non-Western populations.

### Emerging Adulthood Within the South African University Context

Emerging adulthood has been identified as neurologically distinct, with particular capabilities, such as emotional regulation, rational thought, gratification delay and risk calculation yet to fully form (Nelson, 2021; Steinberg, 2017; Taber-Thomas & Perez-Edgar, 2016). Capturing the socially-constructed expectations of the young adult generation (adult children and students), the period of emerging adulthood is characterized by ambiguity about the attainment of adulthood and matters of responsibility and autonomy (Arnett, 2014). Over the past two decades developmental tasks, representing both opportunities, and challenges unique to this stage of life have been identified (Ohannessian, 2021a). These include identity exploration, instability, self-focus, feeling in-between adolescence and adulthood, and viewing the future as full of possibilities (Arnett, 2007). The ‘in-between adolescence and adulthood’ feeling is particularly pronounced for university students as their financial and family responsibilities are usually limited. This makes possible a space for self-focus in which to determine their needs, desires and aspirations (Skulborstad & Hermann, 2016). This period is generally regarded as a period of increased optimism with a sense of possibility for the future (Arnett, 2007).

Considered by some as an extended period of adolescence, much like Erikson’s (1968) stage of identity versus identity confusion, emerging adulthood is a time when individuals prepare for and explore both career and social identities, moving away from parents or caregivers towards peer relationships for identity exploration and a sense of belonging. There is heightened need for social connection and acceptance from peer groups, and an increased sensitivity to peer influence (Andrews et al., 2020). It is a time of instability as they adapt to changes in the transition from high school to university, in some cases move from the family home into university residence or independent housing (Arnett, 2007). It is notable that attrition rates are highest in the first and second years of university, demonstrating the difficulty of this transitional period for many students (Halliburton et al., 2021). Tertiary education attrition rates are notable in the South African context, with approximately one in three university students and one in two technikon students dropping out (Letseka & Maile, 2008). Significantly, however, issues of language, an articulation gap between secondary and tertiary education, and a lack of affordability have all been implicated, rather than issues of social adjustment (Murray, 2014; Mutepe et al., 2021).

Critiques of the theory of emerging adulthood pertain to the rigidity of a stage model of development and questions regarding the cross-cultural and cross-contextual applicability of the theory. It should be noted that the theory of emerging adulthood appears to be more applicable in developed countries. However, as Nelson (2021) reminds us, the theory was developed with mindfulness of the necessity to consider unique and variable normative trajectories and timings through this developmental period. In developing countries, the theory has been found to be more applicable among urban rather than rural young adults (Arnett, 2015). Although the validity of the emerging adulthood construct in Africa has been previously demonstrated (Naudé & Piotrowski, 2022; Obidoa et al., 2019), more research within these populations is required (van Breda & Pinkerton, 2020).

The COVID-19 global pandemic has been identified as potentially having unique and particularly detrimental outcomes for emerging adults (Farris et al., 2021; Glowacz & Schmits, 2020; Shanahan et al., 2022). In a period already marked by instability, “the added uncertainty and unpredictability of the global outbreak and related social disruptions may negatively impact the mental well-being of this vulnerable population during a critical developmental stage” (Farris et al., 2021, *p*. 463). Long-lasting effects on emerging adults have been predicted, related to the emotional consequences of isolation (Ohannessian, 2021b).

The South African government’s response to the COVID-19 pandemic in March 2020 was a National Lockdown that restricted movement from home and enforced social distancing, closing schools, universities and all non-essential services. Stages of the Lockdown eased and returned in line with COVID-19 reported case numbers until April 2022 and limited numbers of people in indoor spaces. Schools and universities shifted to online learning for the most part of 2020 and university undergraduate classes (due to their size) also remained online for most of 2021. In 2022 universities returned to on-campus classes and used hybrid models of teaching. The lockdown was considered severe, and has had enormous economic and social costs (Seekings & Nattrass, 2020).

## Methods

### Research Design

This qualitative study used a cross-sectional survey design to explore student experiences of the COVID-19 pandemic with respect to their perceptions of its impact on their physical and emotional health, how their experiences may have changed over the course of the pandemic, and how they perceive the pandemic and its associated restrictions may have influenced their development as young adults. A qualitative approach was chosen as it “highlights intricacies of the human experience and so is particularly suited to unpack the nuance and depth of emerging adults’ lives” (Schwab & Syed, 2015, p. 397).

### Data Collection

An online survey was conducted. Undergraduate students from the Faculty of Humanities at the University of the Witwatersrand in Johannesburg, South Africa, were invited to participate. Participants were told that researchers were interested in their experiences during and after the COVID-19 pandemic. Participation was voluntary and anonymous. Participants received no compensation for participation in the study. The choice to focus on undergraduate students was motivated by findings that during quarantine, being an undergraduate (a freshman), as opposed to a postgraduate student (a senior), was associated with self-reported decreases in mental health (Hall & Zygmunt, 2021).

Three open-ended questions were posed:

1 Briefly describe your experience of the COVID-19 pandemic and the impact it has had on you - physically and emotionally.
2 Briefly describe how your experience changed since the beginning of the pandemic to now.
3 Looking back, do you think the COVID-19 pandemic and associated restrictions have influenced your development as a young adult? Please explain.

### Participants

The sample age ranged from 18–54, with the vast majority of participants qualifying as emerging adults (falling within the 18–29 range); nine participants were ‘mature’ students - 30 years old or above. The majority of the sample identified as being Black (48.57%) and female (85.7%). 80 participants were first year students, 26 were second year students and 34 were third year students. It should be noted that the majority of the first-year group was completing secondary education (Grade 11 and 12) during the height of the pandemic, and entered university as restrictions began to ease, in-person classes resumed and campus life returned to ‘normal’. Table 1 provides an overview of the sample for each undergraduate year of study.

**Table 1.** Gender and Race Demographics of the Participant Group.

Demographic Factors		First year *N* = 80		Second year *N* = 26		Third Year *N* = 34	
		Frequency	Percentage	Frequency	Percentage	Frequency	Percentage
Gender	Female	66	82.5	23	88.5	31	91.2
	Male	11	13.8	3	11.5	2	5.9
	Non-binary	3	3.8			1	2.9
Race	Black	41	51.2	14	53.8	13	38.2
	Coloured*	6	7.5	2	7.7	4	11.8
	Indian	15	18.8	3	11.5	9	26.5
	White	17	21.3	6	23.1	8	23.5
	Other	1	1.3	1	3.8		

*The racial designation Coloured is an ethnic group native to South Africa with multiracial heritage over generations. Ancestry is mixed and includes Khoisan, Bantu, European, Austronesian, East and/or South Asian.

The responses from the nine students above the age of 30 were left in the sample, as the study was interested in the experiences of all students. The ways in which these students’ experiences diverged from those of the younger students was noted in the analysis of results.

### Data Analysis

Reflexive thematic analysis was used to code the data, and responses relating to the focus of the paper – namely students’ mental health challenges and perceived developmental fall-outs – were identified and grouped into themes (Braun & Clarke, 2019). This was conducted for each year group. Themes that were prevalent in approximately a quarter of responses per year-group were included. After themes were corroborated by a second researcher, the themes that emerged from each year group were compared and contrasted for any differences in experience, and regrouped into larger meta-themes to capture the experiences of the entire group. Differences in the narratives between year groups were considered in light of the fact that each year group was affected differently by the COVID-19 pandemic and the National Lockdowns in South Africa. Third-year students had their first year significantly disrupted by the start of the pandemic in March 2020. The second-year cohort had their final year of school disrupted, but had entered with a year of online-learning experience and an expectation that university classes would be online. First-year students had entered after the Lockdown was lifted with expectations of in-person classes and their final years of secondary school having been completed online. Themes were then interpreted through the lens of the theory of emerging adulthood, which provided a useful way of understanding how disruptions to expected developmental opportunities appeared to result in negative mental health consequences, and how the mental health consequences of the COVID-19 pandemic disrupted expected experiences and strivings of emerging adulthood. The researchers on this study range from early to mid-career (two of the researchers fall into the emerging adulthood age group themselves) and the group analysis approach allowed for useful reflexive and collaborative discussion on the analysis and write-up of the findings. The trustworthiness and credibility of the analysis was ensured through a thorough documentation of the process of the research, through double coding, and through the provision of direct quotes to evidence interpretations of the data (Stahl & King, 2020). The direct quotes included in the findings are indicated per year group: first year (FY), second year (SY) or third year (TY).

### Ethical Considerations

Ethics clearance for the study was obtained from the Human Research Ethics Committee at the University of the Witwatersrand. Contact details for free university counselling services were provided to all participants, as due to the anonymity of the study no individualised feedback could be given.

## Findings

Overall, there was a significant overlap of themes between year groups. Reported impacts of the COVID-19 pandemic on these students’ physical and emotional health are described below, with particular attention paid to on-going sequelae. Students reported continued high levels of anxiety and depression. Social isolation emerged as having had a significant effect on students’ perceptions of their social development and adjustment to university. The quotes included below are recorded verbatim from the various student narratives.

### The Impact of COVID-19 on Physical Health

A small proportion of students across all years reported poor health due to the effects of the coronavirus directly: “I had COVID 3 times so far. The most recent time I am still feeling ill from and that was about 3 mon ago” (FY); “After getting COVID, my immune system is severely compromised and I have severe aftermath symptoms” (TY); “I have been overly concerned about my health and the possibly recurring symptoms of my condition”; “I am physically weaker and more prone to illnesses” (TY); “I also had Covid… and since then I have not regained normal smell” (SY).

Most, however, reported indirect effects on physical health through the National Lockdown, such as weight loss/gain. While a minority of students lost weight: “I lost a lot of weight, and lost my appetite during the heat of the pandemic in 2020” (FY), weight gain was the most common lament across year groups. Most students associated their weight gain to the lack of opportunity to exercise during the National Lockdown and subsequent decreased physical activity and lack of motivation: “As we were ordered to stay indoors I gained too much weight” (FY); “So because I wasn’t on campus and with all the restrictions, I was barely moving around. Most days I went from my bed, to the kitchen, to the bathroom, and back to my room again. Movement was quite restricted” (TY); “I gained weight that I still haven’t been able to lose, I didn’t exercise at all, I was constantly fatigued” (TY); “Physically I am not as fit as I used to be” (TY).

Many students recognised that anxiety and low mood contributed to their weight gain: “I have gained weight, due the stress of the pandemic and the lack of exercise” (TY): “I got extremely lazy…and put on a lot of weight in that period…I was demotivated and sad” (FY); “I have gained so much weight that I am struggling to lose currently…I have been diagnosed with clinical depression and I experience a great deal of sadness lately” (FY); “Physically I ate my feelings and gained a lot of weight but I am trying hard now to lose it” (SY). Students appeared to understand the reciprocal relationship between mood and exercise, but many appeared unable to muster the necessary motivation to exercise, despite awareness that feeling better about their bodies may lift their moods: “I had no time or interest in physically exercising which would have probably helped a lot with my stress levels and anxiety” (FY). There was also an awareness of how weight gain affected mood: “I had gained a little bit of weight and I became very uncomfortable around people and dreaded socializing. My self-esteem was negatively affected” (FY); “During the pandemic I picked up weight…my confidence went right out the window” (FY); “My weight changed and that lead to a lot of emotional strain on me because I am very self-conscious about how I look and social media opinions do not help” (FY); “[My weight gain] has impact [ed] my emotional wellbeing, as I feel that I have lost confidence and my self-esteem has taken a knock” (TY). For a few students, this then resulted in unhealthy compensatory behaviour: “I developed unhealthy eating habits where I would starve myself” (FY).

### The Impact of COVID-19 on Mental Health

#### Fear and Anxiety

Levels of anxiety were reported as high in the initial stages of the pandemic with words like ‘fearful’, ‘uncertain’ and ‘stressed’ featuring commonly in the narratives: “It was difficult not knowing what the future looked like” (TY); “During COVID-19, everything was scary. There was a ton of uncertainty” (TY). For some students this uncertainty seemed to contribute to extreme feelings of fear: “I suffered with severe anxiety” (FY); “emotionally I had outbursts thinking the worst could happen” (FY); “I was stressed and anxious with constant breakdowns” (FY). For the majority of students, however, this initial anxiety eased as time passed and restrictions lifted, but for a portion of students, the elevated anxiety has remained: “I feel more anxious and artificial. Not really in touch with myself, the way I was before”; “It has sparked within me a wariness of the world”; “The COVID-19 pandemic has made me very anxious and I still notice it today, there are many things I fear which I had not feared prior to the pandemic”; “My anxiety seems to be getting worse” (TY); “I was not as anxious as I am now and it seems like it is not a trait I will lose soon” (TY). For some, the resumption of pre-COVID-19 activities has been problematic:

The emotional effects of the pandemic have really affected me. I was never really scared of being home alone but during the pandemic when my parents went back to work and my sister went back to school, I would get a lot of anxiety or I'd have daily panic attacks. Going back to my normal routine - things like attending church give me panic attacks (TY).

During the height of the pandemic, much anxiety appeared to involve fear of illness and potential loss of loved ones: “Emotionally it drained me as I was anxious about loved ones consistently” (FY); “During the first wave of the pandemic, I remember having to go to the store and shaking at the idea of having to interact with other people. I did not leave the house for another fo [u]r months after the incident. I was constantly worried and stressed about my family” (TY); “My Covid-19 experience was quite traumatic, the rate of death and infections was alarming…I was scared for my life and loved ones’ lives. It was horrendous” (FY). In a portion of the students, the development of reactive responses, emotions and hypervigilance as a consequence of COVID-19 appears to have remained: “I lock myself in” (FY); “I am more alert and tend to over stress” (FY); “a lot more aware of health and hygiene than before” (FY); “it left me much more on edge” (FY); “The pandemic made me quite sensitive to hygiene and I now carry sanitiser everywhere and wear a mask if I’m not feeling well just to keep others safe, even if it’s just a flu” (FY). These persisting fears seemed most marked amongst participants who had suffered losses: “July 2021 I lost my father to Covid (the delta variant). This has made a huge impact on my emotions obviously. I have an irrational fear of death and I am scared that the people around me will die” (SY); “[I have] the irrational fear of people dying around me. Which makes me hesitant to connect” (SY). For other students, however, this anxiety has subsided as case numbers have lessened and restrictions have lifted: “At the beginning I was constantly afraid, overly cautious, and had a lot of anxiety. These feelings have mostly subsided” (TY); “I am less panicky now since my family and I are fully vaccinated” (TY).

#### Grief

A number of students reported the loss of a loved one due to COVID-19: “the pandemic had a huge impact on my life. I had lost loved ones from it” (FY); “Emotionally it has left me numb with all of the loved ones I lost” (FY); “During the Covid 19 pandemic I lost my brother. This has kind of like disturbed me emotionally because I would normally cry myself to sleep” (TY); “The past 3.5 mon I have lost two close friends and it has emotionally impacted me “(SY); “I lost my grandma through Covid19 it was the worst experience ever 
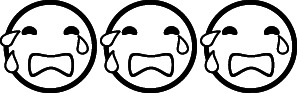
 till today I'm ’till not okay” (FY); “I lost my mom during this period…emotionally it has done some real damage because everything changed rapidly” (SY). For a portion of these students, the grief from these losses was notably raw and the devastation experienced was clear:

I lost my father to Covid, I don’t think I will ever get over that. I did not want to talk to anyone or see anyone because I didn’t see the point of it and didn’t want to bring up the pain. I've become much more introverted and closed myself off to most people. Emotionally I was a wreck, my father passed away overseas, due to Covid restrictions on a lot of countries I could not even go overseas to see him, the entire ordeal was extremely devastating for me and I feel I will never be the same because of it. My dad and I were very close, till today it feels like yesterday to me that he passed and it’s hard for me that the world is just moving on like nothing happened. I’m slowly learning to go on with life but he is forever close to my heart. Activities and day to day things just don’t feel the same anymore, I've lost interest in almost everything (SY).

Losses did not only involve the death of a loved one; in some cases, COVID-19 had resulted in disability: “My parents were really ill and my mother has been left disabled after having Covid, the Delta variant” (FY); “My granny…suffered a mild stroke as a result of COVID-19. She didn't ’ose any capabilities per se but she definitely became weaker than her usual self” (FY); “Emotionally, we suffered a massive heartache and now living with someone who is mentally inapt*(sic)* due to Covid emotionally it was tough” (SY).

#### Financial Stress

In addition to the strain of disabled family members, often the losses experienced also entailed practical and financial difficulties: “My entire family had covCovid besides me so I was running the household and that added both physical and emotional strain” (FY); “Losing my grandma meant I need to find a relative to stay with as we were living alone the two of us” (FY); “The pandemic was hard for many people including my family and it broke my heart to see them suffer like that physically and mentally” (FY); “I'm ’ecoming too emotional as I write this because so much has transpired. I have to support some of my family members who lost their employment while I also don't ’ave enough to spare. Without God's ’elp, I don't ’now where I would be right now” (SY).

#### Loss of Freedom

Another kind of loss that emerged pertained to the National Lockdown and the associated restrictions on movement. Many students reported feelings of being trapped in their homes: “I felt trapped in my house. I felt irritable and fatigued most of the time” (FY); “I felt trapped and stagnant and unmotivated” (FY); “It felt as if my life was placed on pause” (FY). This extended to feeling ‘robbed’ of time and experiences normative for early adulthood: “I feel less connected to people, I feel like I've’lost time and opportunities for connection that I can't ’et back” (SY); “I do however think that as a student I missed out on different social events and experiences that would have been good memories to look back on when I am older” (TY). A strong sense of lost or dashed expectations emerged:

For [those who] began their first year at university in 2020, I think a lot of people have the expectation that their university years are when they will make new friends and potentially meet their future spouse. I think that those who were only able to experience about a month on campus before we went into lockdown missed out on the opportunities to begin and foster crucial relationships (TY).

Interestingly, this sense of having lost out on normative experiences appeared most prominently amongst the first-year cohort, the majority of whom were in Grade 11 and 12 during the pandemic: “Much of my high school was during covCovidd I feel like I didn't ’et to experience it as I should have” (FY); “I wasn’t able to experience normal things one would in high school like parties and sport” (FY); “I feel like the pandemic took away ‘the best years of my life’…so I do think it did take certain key development moments away from me” (FY); “I was doing my matric when the pandemic started so I felt rather cheated by life and I was extremely unhappy during that time” (FY); “I was not allowed to go out and socialise during one of the most social years. Many experiences were different- maric dance and going out with friends” (FY); “The more time that went by the more robbed I felt of my late teenage years. I also got adapted a very toxic mindset and got depressed” (FY); “I was demotivated and sad, I was angry that 2 two my most important years as a hockey athlete was taken away and that my most important academic year had to be complete online with no assistance at all” (FY). The loss of normative high school experience extended into an experience of loss with regards to beginning university: “Emotionally, I feel as though the pandemic as ripped away certain experiences of my life such as the university experience and being around new people and making new friends which has made me feel lonely at times” (FY).

#### Depression

This loss of normative experience, together with financial losses, and the loss of loved ones appeared to have resulted in depression for a portion of the participant group across cohorts, with notable themes of withdrawal, a sense of disinterest and a loss of motivation as a consequence of COVID-19 disruption: “It was a difficult period for me. I was diagnosed with depression, anxiety and burnout and Bell's ’alsy at the same time” (SY); “Emotionally I became significantly more depressed over time due to the isolation” (FY). Many students reported continued low mood and a decrease in motivation: “Before the pandemic I was happier and more energetic. I had a lot of motivation but this is not the case anymore” (TY); “I am no longer interested in jogging or going to the gym as I used to before the pandemic. I am in bed all day if I have nowhere to go to. I can't ’ven get up to clean my space” (FY); “I have become very introverted and closed up and lost majority interest in everything in life in general” (SY); “I have lost interest in the things I would mostly do to cope with life” (FY); “I am experiencing a lot of things differently now. I am no longer enthusiastic about a lot of things, gym, work, church, friends, social life, social media etc.” (SY); “I often feel low in energy and demotivated. I'm ’ore withdrawn and depressed than I used to be before because I'm ’lways indoors” (SY); “Generally, my mental health fluctuated quite a bit over the pandemic. It was hard for me to fully process what happened…I also sunk to some of the worst places I've’been mentally” (TY).

A clear theme of hopelessness emerged in relation to the future: “From feeling hopeful and determined to feeling insecure and stagnant. I'm ’ot as optimistic about life as before” (TY); “At the beginning of the pandemic I had a more positive outlook on life and more drive to work towards something, now I feel as though life is a bit of a drag and nothing makes sense” (TY); “The pandemic in general has left me feeling extremely fatigued and with feelings of hopelessness” (SY); “I now have a more pessimistic view of the world and have a hard time believing that things will get better” (TY); “The pandemic has impacted me in a very negative way both physically and mentally. It has filled me with feelings and dread and uncertainty about the future” (FY).

Part of the hopeless narrative centred on uncertainty about the future that the shock of COVID-19 appeared to have brought into sharp relief: “I am less hopeful about the future as anything can happen anytime (FY); “Stress more about my future because nothing is really predictable”; “I just live in a state of anxiety now though because I expect for there to, at any given moment, arrive a pandemic that could change our reality as we know it (FY). This uncertainty also featured fears about the economic sequelae of the pandemic and potential future employment: “I started to think a lot about my future, whether I have chosen the right career as people lost jobs during this period (FY); “Also, in the beginning of deciding what one wants to do with their lives professionally, the impact of the pandemic left much uncertainty and anxiousness for the future” (FY); “I am feeling so depressed about our petrol (gas) price and the increased cost of living…which places strain on me financially” (SY); “The pressure to become successful and be able to provide for myself and my family increased immensely” (FY). There was recognition that this feeling of hopelessness was a shared sentiment:

There also seems to be a cloud of stress and anxiety that has plagued the whole country. With everyone being financially impacted by the pandemic, it seems the average South African has gone down in happiness and the isolation has made everyone that bit less friendly (FY).

The hopelessness was linked to a greater sense of isolation attributed to the National Lockdown: “During the pandemic I felt very secluded and cut off from the world and I felt hopeless at some points in time” (TY). Feelings of isolation emerged as a strong theme in the data and was often linked by participants to increased social anxiety.

#### Isolation and Social Anxiety

Students felt alone and reported the lockdown to have negatively impacted their friendships: “During the pandemic I felt cut off from my friends” (FY); “It was a very isolating experience for me” (FY); “some friendships became strained as we were unable to interact in a way that wasn't ’nline” (FY); “I also feel like I've’drifted from people who were important to me because we didn't ’ave contact during covCovidFY). Many of the students associated their feelings of isolation with mental health challenges: “A[n] almost constant state of solitude and withdrawal. Feeling distant from myself and feeling as though I don't ’ctually know myself. Emotionally not so good” (FY); “I began to isolate more, my anxiety spiked” (TY); “During the hard initial lockdown, I found it hard to cope because I could not see and interact with other people” (SY); “Being alone is very stressful on a physical level, this is something I only understand now” (SY).

Interestingly, however, despite insight into the fact that isolation was negatively impacting their mental health, many students reported continued lack of social engagement even after restrictions pertaining to social distancing were lifted: “Still feel isolated to some extent” (TY); Since lockdown I stopped being social, so much that I would avoid being around people” (FY). Most students appeared to understand this as related to them having adjusted to less social interaction and a resultant lowered motivation to engage socially: “I was a lot more outgoing before the pandemic and really enjoyed a lot of activities however I no longer feel an urge to do these activities anymore” (FY); “I've’found that I'm ’omewhat more reclusive and less enthusiastic about going out and doing things in person. I now prefer online school/university and I feel as though it may be an effect of the lockdown” (FY); “although I've’become more introverted I see things very differently now and society in general” (SY); “I've’become more of an introvert and prefer to deal with my issues by myself which is something I adopted during the pandemic” (FY); “I've’grown comfortable in my solitude now, but then I hated being so alone, now I like being in my own company” (FY). Some students, in fact, appeared to find less social interaction preferable: “I want to go back to level 5. Everything was easier and more peaceful and I didn't ’ave to see anyone or do anything and I liked that. I focused on myself a lot and it was good for me” (FY). Others, however, experienced this shift as negative: “I went from being a reserved person to being a totally closed off person” (FY); “I believe that it has influenced me to a certain extent as I am more "re“lusive" and don't ’eally go out anymore, even though all restrictions have been limited. This makes it more challenging to meet people and make friends in university as I am quite reluctant to leave the house” (FY).

Others attributed the continued decrease in social interaction post Lockdown to heightened social anxiety. Ironically then, the lifting of restrictions was also experienced as anxiety-inducing: “My anxiety is more constant, and I tend to avoid people more often as now I feel drained when I'm ’round people whereas before I was able to find some joy in social interactions” (FY); “The pandemic and extended period of isolation has made me more shy and I experience social anxiety more than in the past” (FY); “It has kind of limited my abilities to be sociable as I used to be and I find it difficult to leave the house sometimes” (FY); “The pandemic put me in my shell too much, having to isolate for me didn’t end with the pandemic as I still do” (FY); “I think the pandemic and restrictions increased my social anxiety and introversion, I have enjoyed staying inside my house and not needing to be anywhere” (SY).

Notably, in line with the broader theme of social anxiety that appeared in the larger group, the most commonly reported symptom to have worsened over the course of the pandemic in students with existing mental health issues, was that of social anxiety. Any progress in managing their social anxiety that had been made prior to the pandemic was felt to have been lost, as these symptoms remained heightened:

I believe that Covid worsen[ed] my social anxiety. As I have to start afresh to cope with being around people (FY)

I feel like my social anxiety has gone back to being quite bad and I feel like all the progress I made with being able to be unintimidated by large crowds has gone away and I am once again anxious and scared around large groups of people especially if I don't ’now them, this has made attending classes at Uni a lot harder as I know almost no one at my Uni and none of my previous friends go to Wits. This means I have yet to make any new friends and this upsets me as I thought I had learnt to deal with this and could make new friends, now it feels like I’m back to square one (FY).

Of note is that the theme of social anxiety featured particularly strongly amongst the first-year cohort. This reported effect of the national lockdown with its associated restrictions on movement and social interaction appeared more pronounced in the first-year cohort. During their final 2 rs of high school, they appeared to find COVID-19 particularly disruptive to their social relationships and social confidence:

When school began again in person, my social anxiety had skyrocketed. I began to be stressed in situations I'd never normally be anxious about, specifically social situations. I felt very insecure within myself and that was the year where my grades had been the lowest they ever had (FY).

The start of the COVID-19 pandemic was a disruptive time on my life in different ways… I had issues with my emotional wellbeing and as a result I didn't ’ope as well as I had hoped in my final years of High School. I felt trapped, isolated and anxious, which are all normal reactions, but I felt unable to do what needed to be done to cope; this strained relationships in my life because I didn't ’nderstand what I felt. Emotionally, beyond social and general anxiousness, I had a tough time bouncing back (FY).

Many responses from the first-year cohort suggested that the lockdown had in fact impaired their ability to function socially: “after the pandemic, it's ’een harder to get along with people and make new friends” (FY); “The pandemic has definitely taken away my social traits, I feel like I have no clue how to act in social settings anymore and being in social settings now just make me spiral and panic” (FY); “Emotionally I feel I am no longer able to function in social environments” (FY); “restrictions of going out which made me anti-social/socially awkward” (FY). This impairment in social functioning was understood to be a common experience across peers: “I have noticed that a lot of people my age have forgot how to socialise or become extremely awkward in conversation” (FY). A clear sense that an emerging skill related to social competence had been damaged during the pandemic was revealed:

More than anything, it has been a big detriment to my social life. I struggle to make friends and I find it nearly impossible to get into a relationship because I haven't gone anywhere or met new people…I feel like the isolation may have damaged my ability to meet new people and I struggle to cope with the fact that I may feel this lonely for years to come (FY).

I have felt less isolated since I could see my friends and teachers in High school again but now that I am at Wits I know no one and I have been having trouble with the large groups of people, to the point where they stress me out instead of being a comfort. I feel like I have to relearn a skill that took me years to learn and I feel like an old problem has come back (FY).

A felt sense of regression in social skills was evident, with students feeling developmentally younger and less self-reliant than they imagined they would feel when beginning university: “I feel as though I have unlearnt all how to cope with meeting new people and as though I am back in my childhood when I found it hard to cope with strangers and lots of people which I don't ’now” (FY); “My social skills that I currently have would have developed a few years ago” (FY); “My social skills have definitely been impacted, and my ability to reach out to others has weakened” (FY); “I believe it has made me more anxious, reliant on people and reliant on social media or my phone in general” (FY); “My social anxiety has worsened back to where it was in middle school and I now can't ’ake friends with people on campus as they make me anxious” (FY).

This sense of regression was also apparent in the other cohorts, and was understood as a response to the fear and anxiety associated with COVID-19: “It[CO VID-19] limited me from creating relationships with other people and has made me socially anxious. I am scared of starting relationships with people. I prefer being online than being in physical contact with people. I am now a socially awkward person” (TY); “I often get anxious when I'm ’n a big group and people are not socially distanced which is not something that I would feel before” (TY); “I have not had the chance to develop my social skills, through my university experience and so I navigate campus and making friends the same way I would if I was a first year and the thought of it more often than not, scares me” (TY).

### The Impact of COVID-19 on Development

While most prominent in the first-year cohort of students, across all cohorts there was a strong theme that the COVID-19 pandemic and associated restrictions had resulted in delays in development: “[COVID] has sheltered me in many ways” (FY); “I feel if COVID did not happen, I would be a lot more equipped for the outside world” (FY); “I think that it slowed down my development in the sense that I would have had more opportunities if the pandemic had not happened” (FY).

A strong sense of experiences or ‘rites of passage’ crucial to development having been lost to the pandemic emerged: “I was in a way restricted from experiencing certain rights of passage, that teenagers usually experience” (FY); “I think the reason for us changing is attributed to spending 2 years that are vitally important for our development and growth in a more isolated manner” (FY); “I have missed out on a few coming of age events and rights*(sic)* of passage” (FY) “I think it took away a lot of needed and valuable events/ siuations academically and socially” (FY); “I am worried about key experiences I may have missed out on” (TY); “There are steps I think I missed out on because of the restrictions of the pandemic. Being stuck at home did nothing good for my development” (TY).

This loss was acknowledged as having lessened opportunities to learn independence: “It made a university experience different from what others had. I could not experience the fun times or even making new friends. I could not learn independence or get into meaningful relationships” (TY). It was felt that the increased isolation and a lack of experiences had restricted their development and perspective on life: “I'm ’iewing life at a limited lens” (FY).

A subjectively felt delay in the development of crucial social skills featured most strongly. The lack of opportunities to socialise and expand their social capacities were lamented and this was felt to have negatively influenced their confidence and ability to make new friends, compounding their social deficits: “I did not get the chance to fully develop my social skills that are needed to create relationships with others” (FY); “The Covid-19 restrictions made it much harder to develop a social life and people often forget how to socialize or be more open towards people who are different from them” (FY); “Lockdown restricted me from participating in sporting activities for my school and other social events like speech contests. This really impacted my social skills as well as my confidence” (FY); “I have become more reserved and unable to communicate myself well” (FY).

These delays in social skills were then felt to have impeded adjustment to university life: “If I had maintained the level of social comfort I had pre-pandemic I feel like I would have been able to adapt to Uni life a lot easier” (FY); “I used to be quite adaptable and very sociable but now I find it difficult to socialise and large groups of people are extremely intimidating” (FY); “Covid hit when I was in 1st year and so I missed out on many of the 1st year experiences. I think I struggled the most with not making strong enough relationships at the beginning of 1st year to continue them during Covid so many of my friends were still from high school and I found it difficult to reach out to people” (TY).

## Discussion

Although the first two2 years of university have been identified as an extended period of transition with normative psychosocial adjustment challenges (Conley et al., 2020), for many South African students adjustment to university is particularly challenging, given high rates of first generation university attendance, significant financial challenges and high levels of mental health challenges (Bantjes et al., 2019; MurMurray, 2014; Mutepe et al., 2021While this study adds support to the international literature documenting the significant levels of distress in the young adult population during the pandemic (Chen & Lucock, 2022; Copeland et al., 2021; Li et al., 2021; Nurunnabi et al., 2020; [Bibr bibr33-21676968231180960][Bibr bibr44-21676968231180960]it is clear from this study that the COVID-19 pandemic with the preventative quarantine and lockdown measures that were instituted appear to have had further profound impacts on South African student well-being. With the data having been gathered after the National Lockdown had lifted, these participants had had time to reflect on the potential consequences of their distress for their development and adjustment to university.

Farris et al., (2021) warned that emerging adults, who are already experiencing instability associated with their life stage, may be particularly vulnerable to the unpredictability of the COVID-19 pandemic and the uncertainty related to associated social disruptions. This study confirms that beyond the disruption of the transition to online learning and for some, relocating back home, the broader global disruptions, including fears around the progression of the virus, and subsequent economic uncertainty and instability, appeared to have increased students’ anxiety. Many students appear to have withdrawn, become disinterested and lost motivation as a consequence of COVID-19, remaining isolated and separate from others. This, together with decreased sense of hope for the future, and notable complaints of weight gain, suggested high levels of depression in the participant group. There was an increased sense of wariness about the future as well as uncertainty about their own self-worth and place in an unpredictable world, with a portion of students reporting a continued negative outlook on life.

A strong sense of loss emerged from these student narratives, which, beyond the grief in response to loss of loved ones, included the loss of highly anticipated social experiences, imagined ‘future selves’ and the freedom to explore, which dashed expectations. In a developmental period where there is an implicit social imperative to “have a lot of fun, be carefree, and do many adventurous things before becoming an adult” (Nelson, 2021. *p*81), this extreme response of feeling ‘robbed’ is understandable. Concerns about the potential loss of future opportunities due to local and global economic ramifications also contributed to a sense of lessened ‘possibilities’. There was also a loss of opportunities for self-focus – an important aspect of this developmental stage. Opportunities for self-focus, which have been theorised as necessary preparation time (Nelson, 2021) were clearly forestalled for some students. Strong themes about anxious preoccupation with the well-being of loved ones emerged, alongside accounts of having to take on more adult responsibilities such as running a home or contributing financially after caregiver illness or loss. The pressure of having to be more ‘grown-up’, but without the necessary capabilities, experience or confidence appeared overwhelming for some students. Loss of opportunities for self-focus and heightened instability seemed to have disrupted students’ appropriate developmental need to view the future as full of possibilities.

Notably, as in Ewing’s (2022) ….sample, these South African students experienced social isolation as particularly challenging. When considered in light of emerging adulthood as a time of identity exploration, this strong response is rendered more understandable. During a time of identity exploration, when young people are working to find meaning in their work and in their relationships (Arnett, 2014 repeated National Lockdowns of varying levels and the transition to emergency remote teaching made this less possible and disrupted identity development processes that should occur during this developmental period (Arnett, 2014 The data suggested that some students felt that exploration was forcefully shut down during lockdown, while others experienced the ongoing stress, anxiety, and depression as a result of the pandemic as interrupting much anticipated identity exploration through the formation of relationships with others (romantic or otherwise) during the pandemic. For some South African students for whom university represents the potential to move up in social class and ‘escape’ backgrounds of poverty, the loss of the chance to be in residence and explore alternate social groups and lifestyles was profound and may have contributed to feelings of being trapped – perhaps not only in the family home, but also in the familiar day-to-day financial stressors and strains of living associated with lower socioeconomic contexts. The extreme distress at increased isolation evident in the narratives appeared linked to elevated anxiety and lowered mood, and is associated in the narratives with lowered levels of self-confidence and less ability to connect with themselves emotionally, let alone with others interpersonally. Fewer opportunities for social connection appear to have left a large portion of this student population feeling socially inept and disappointed at the loss of opportunities for exploration of self in new relationships and environments.

While students from the second and third year cohorts did raise concerns over having had less opportunity to make friends on campus, of significance was the response from the first-year cohort. While the second and third year students had experienced university for the most-part online, first-year students had entered after the National Lockdown was lifted with their final years of secondary school having been completed online. While second- and third-year students bemoaned the lack of opportunities to socialise, the first-year cohort appeared acutely aware of their lack of opportunity to have developed social skills they felt they needed in order to thrive and grow at university. Although they had begun university at the beginning of 2022 when social restrictions had eased and they had, in fact, been able to come to campus and attend some classes in person, they felt that their social skills were underdeveloped and struggled to use the opportunities provided for social interaction. Their levels of self-confidence in social settings appear to have decreased, suggesting that the last two years of high school may indeed be vital for the development of social confidence and the skills required to make new friends at university; this group of students missed their chance to be ‘the big fish in a small pond’ before joining the much bigger pond of university. Indeed, Halliburton et al. (2021) warned that the ongoing pandemic may impact emerging adults “during a critical developmental stage that shapes the way individuals mature into functioning adults” (p.4*p*3). Correspondence from UN Educational, Scientific and Cultural Organisation (UNESCO) Chair on Health Education and Sustainable Development in the Lancet highlighted that interruptions in schooling as a result of the COVID-19 pandemic demonstrate that school fulfils not only educational needs, but also provides for the meeting of crucial socialisation needs of young people (Colao et al., 2020). This lack of space and opportunity to form necessary social connections, which was highlighted repeatedly in the data, has clearly emerged as having negatively impacted developing social skills. The sense of having ‘regressed’ in terms of social skills that was found in these students’ narratives, makes sense in light of other studies that have previously found a similar sentiment, namely a sense of feeling an ‘identity regression’, in relation to emerging adults who have lost opportunities for independence post having lost employment or needed to move back home (Benson & Furstenberg, 2007; Nelson & Barry, 2005). The continued isolation experienced due to the struggle to reintegrate back into social life after restrictions eased was also found to be a clear source of distress across cohorts. This is supported by studies that have consistently found that perceived social support and social connection with peers is related to levels of psychological well-being and adjustment to university (Conley et al., 2020; Pittman & Richmond, 2008). This highlights the vulnerability of this group in particular, as the levels of depression and anxiety evident in the narratives were high, which in itself would make connecting socially more challenging. Given that the odds of academic failure have been found to be elevated among South African students with mental health concerns (Bantjes et al., 2021), this sentiment is of concern, and signals the need for increased monitoring of this group’s mental health, academic performance and requirements for additional support. It appears that issues of social adjustment post the COVID-19 pandemic may also require consideration, alongside issues of affordability and the articulation gap between secondary and tertiary education in the list of risks to throughput rates.

## Strengths, Limitations, Implications and Conclusions of the Study

The qualitative nature of this study implies decreased generalisability, however, this is offset by the opportunity to gather participants’ subjective reports of their experiences and to identify subtle trends in these reports. It is important to note that students only represent a third of young adults in South Africa, limiting the generalisability of findings to the broader young adult population of South Africa. However, this population is important to study, as significant government resources are spent on the higher education sector in order to promote skills development in the country; low throughput rates are a threat to this investment and barriers to qualification completion are of interest (Ajoodha et al., 2020; Letseka & Maile, 2008).

It is also necessary to note that volunteer bias may have influenced the findings of this study, in that it may have been only particular kinds of students with certain kinds of experiences who volunteered to participate, for example, only those who had especially intense experiences or those who had hoped to access some support.

Overall, the COVID-19 global pandemic was predicted in the literature as potentially having unique and particularly detrimental outcomes for emerging adults (Farris et al., 2021; Glowacz & Schmits, 2020; Shanahan et al., 2022), particularly in relation to the “emotional toll from the stress and isolation” (Ohannessian, 2021a, p. 9). This paper provides insight into some of these ‘detrimental outcomes’, as reported by a group of South African undergraduate university students. Elevated levels of anxiety and depression were reported, which has implications for university management, academic staff and mental health services in terms of planning and provision of support. This is also of significance for mental health professionals who are likely to come into contact with vulnerable members of this emerging adult student population.

Most significantly, a strong sense of having ‘missed out’ on formative and necessary social experiences emerged from the data. This appears to have left many students feeling ‘delayed’ in their development of crucial social skills. This would be an important potential implication of the pandemic to investigate using quantitative, and potentially longitudinal, studies. There is also a need for universities to provide opportunities for and to encourage social participation, in addition to academic achievement, as there are strong links between social adjustment, mental health and academic performance (Ryan et al., 2019). In this sense, the developmental conceptualisation of emerging adulthood as involving a heightened need for social connection (Andrews et al., 2020) was useful and appropriate within a South African student population. The fact that the quality of peer relationships have been found to influence levels of behavioural, emotional and cognitive engagement in learning contexts (Ryan et al., 2019) has important implications for the findings of this study, and highlights the importance of addressing the social anxiety reported in this population.

## Supplemental Material

Supplemental Material - In the Wake of COVID-19: The Developmental and Mental Health Fallout Amongst South African University Students Click here for additional data file. Supplemental Material for In the Wake of COVID-19: The Developmental and Mental Health Fallout Amongst South African University Students Katherine Bain, Tasneem Hassem, Nabeelah Bemath, Victor de Andrade, Sumaya Laher in Emerging Adulthood.
